# Effects of Pre-Treatments on Bioactivity of High-Purity Titanium

**DOI:** 10.3390/ma11050675

**Published:** 2018-04-26

**Authors:** Yaming Wang, Guangxin Wang, Zhi Lu, Wuhui Li, Yanfu Yan, Yongfa Song, Osaka Akiyoshi

**Affiliations:** 1 Research Center for High Purity Materials, School of Material Science and Engineering, Henan University of Science and Technology, Luoyang 471023, China; wym5821@163.com (Y.W.); luzhi02013@163.com (Z.L.); whli@haust.edu.cn (W.L.); yanyanfu@haust.edu.cn (Y.Y.); songyongfa2015@163.com (Y.S.); a-osaka@cc.okayama-u.ac.jp (O.A.); 2 Faculty of Engineering, Okayama University, Tsushima-Naka, Okayama-shi 700-8530, Japan

**Keywords:** microstructure, rutile titanium dioxide, high-purity titanium biological activity, nucleation and growth of apatite, inhibitory effect

## Abstract

Titanium and its alloys are frequently employed in medical and dental clinics due to their good tissue compatibility, including commercially available pure Ti, Ti6A4V, or Ti-15Zr-4Ta-4Nb. Yet, they may behave very differently when in contact with our plasma because of their own chemical composition. The present study was designed to compare the in vitro behavior of highly pure Ti (>99.99%; hpTi) with those of the above titanium specimens when they were subjected to heating in air (HT), H_2_O_2_ and heating (CHT), and heating in air after forming grooves on the surface (GT). Since one of the measures of material-tissue compatibility has been in vitro apatite formation in artificial plasma, like simulated body fluid (SBF) of the Kokubo recipe, the apatite deposition in SBF on their surface and in their grooves were examined in terms of the X-ray diffraction, scanning electron microscopy, and energy dispersion X-ray analysis. The results showed that hpTi was as active in in vitro apatite deposition as the other reference titanium samples mentioned above. Moreover, GT specimens of hpTi induced apatite deposition on the platform of the grooves as well as in the grooves. Therefore, hpTi was concluded to have better activity, and to be clinically applicable.

## 1. Introduction

Osseointegration is an important evaluation of titanium and titanium alloys as implant materials, referring to the spontaneous association between bone tissue and implants [1]. Due to inherent inertia, titanium and titanium alloys cannot be directly connected with bone tissues [2]. Thus, various surface modification methods have been proposed [3,4,5]. Sputtering, sol–gel methods, electrodeposition, chemical, spray pyrolysis, electron beam evaporation, chemical vapor deposition, and micro arc oxidation are commonly used methods. In particular, plasma spraying has been successfully applied in clinical practice. However, these methods cannot be applied to complex shaped substrates such as artificial human bones. One problem is associated with high temperature which can change the shape of the substrate, resulting in thermal stress deformation. Another problem is that film-based bond strength cannot meet the mechanical requirements of human implants.

Having abandoned traditional methods of producing titanium dioxide film, Wang et al. [1] proposed a special specimen set-up and found an interesting phenomenon that apatite was found to deposit on titanium in contact with the bottom of a polyethylene bottle within seven days but not found on the side open to Kokubo’s simulated body fluid (SBF), which was similar in ion concentrations to human blood plasma (pH 7.40 Na^+^ 142.0 K^+^ 5.0 Mg^2+^ 1.5 Ca^2+^ 2.5 Cl^−^ 147.8 HCO_3_^−^ 4.2 HPO_4_^2−^ 1.0 SO_4_^2−^ 0.5 mM) even if extended to two months. Sugio et al. [6] made further improvements on the basis of this finding, and “GRAPE technology” was subsequently proposed by them. Unlike magnetron sputtering, this method does not require large-scale test equipment and vacuum equipment, while the shape and flatness of a substrate is not strictly required.

Although Sugio et al. and his colleagues have made stupendous achievements in exploring the optimum groove size and heat treatment in air, there is no conclusive evidence that the hemispheres observed in their work [7] are apatite. Their later study of Ti-15Zr-4Ta-4Nb [8] also failed to explain the anomalous effects that the apatite deposition ability of Ti-15Zr-4Ta-4Nb oxidized at 400 °C was less than at 500 °C and 600 °C, though the amount of -OH groups decreased with increasing temperature. Increasing the heating temperature resulted in an increasing amount of rutile-type titania. However, this phenomenon does not mean the titania gel with rutile structure would lack bioactivity. Hayakawa et al. [9] reported that the crystal phase of the titania layer on thermally oxidized titanium alloy substrate in air did not always promise in vitro apatite-forming ability. Similar results were reported by Sugino et al. [6]. Cho and Peltola et al. [10,11] thought that not all the Si-OH or Ti-OH groups are equally effective for apatite nucleation except those in a specific arrangement. Similar statements are also proposed by Uchida et al. [12]. While Uchida emphasized the importance of the presence of anatase, he also proposed that not all Ti-OH groups but certain types of Ti-OH groups in a specific structural arrangement, are effective in inducing apatite nucleation. 

In short, questions still remain to be answered regarding in vitro bioactivity of titanium or titanium alloys with different oxide layers. Therefore, this work focuses on in vitro bioactivity of titanium with different pretreatments. To avoid any effects of alloying elements or impure elements, our experiment was done with a high-purity titanium (see Table 1 below). One of our titanium specimens was heat treated at 600 °C for 5 h to completely convert anatase to rutile titanium dioxide. This allows an assessment of in vitro bioactivity of rutile titanium dioxide.

## 2. Experimental

### 2.1. Material, Specimens, and Chemical/Heat Treatment

High-purity titanium with the chemical contents as shown in Table 1 was purchased from Six Nine Titanium (Jintan) Ltd., Changzhou, China. Two kinds of specimens were prepared: Type A specimens had a dimension of 10 × 20 × 1 mm^3^. Type B specimen was 10 × 10 × 3 mm^3^ and machined to provide micro-grooves of 500 µm in depth and 500 µm in width. Prior to further chemical/heat treatments, all specimens were sonicated in acetone for 30 min, and then gently rinsed with distilled water three times. 

One of the type A specimens was chemically treated with hydrogen peroxide at a mass fraction of 30% at 80 °C for 3 h and then thermally oxidized in a muffle furnace at 400 °C for 1 h. The specimens were denoted as CHT (chemical and heat treated titanium) [13,14,15]. Another type A specimen was covered with 50 µm crystalline Na_2_O·2B_2_O_3_ powder (Innochem Co., Ltd., Beijing, China) and directly heat treated in a muffle furnace at 400 °C for 5 h. We denoted this specimen as HT (heat treated titanium). In addition, a grooved type B specimen was thermally oxidized at 600 °C in air for 5 h. This specimen was denoted as GT (grooved titanium). Table 2 summarizes the treatments of the three specimens.

### 2.2. Assessing the In Vitro Bioactivity

**Figure 1:**
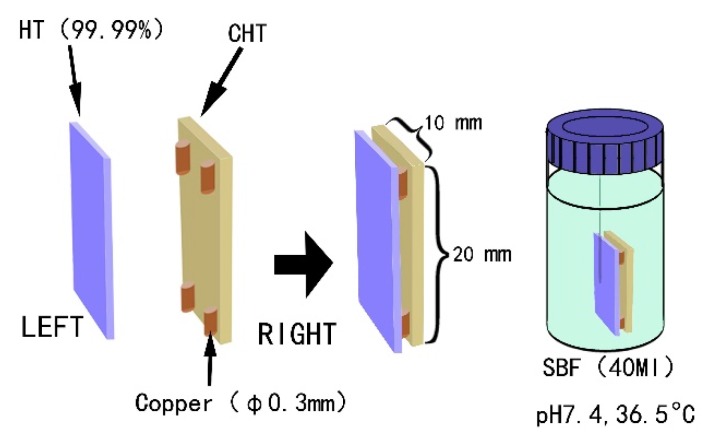
Schematic diagram of the sample setting, where a pair of the specimens was held together at four corners with 0.3 mm copper wires in diameter. A 0.3 mm gap between the two specimens was formed, and the opposing surfaces were denoted as contact surfaces [9].

All treated specimens were immersed in SBF [16,17] and kept at 37 °C for either 5 days or 14 days. CHT and HT specimens were placed in a polyethylene bottle containing SBF together with 0.3 mm copper wires in between as spacers. Thus, there is a 0.3 mm gap between the two specimens (see Figure 1), following Hayakawa et al. [9]. Note here that copper pieces were used instead of Nylon^®^ (New York City, NY, USA) ones. GT specimen was so placed in another polyethylene bottle that the grooved surface was facing down the bottom of the bottle. Once the scheduled time had been reached, the specimens were removed from the SBF liquid and kept in a dry oven at 60 °C for further examination. 

In general, both the rate and the amount of apatite generation can be used as a criteria for judging the bioactivity. To gain related information, a selected specimen surface was examined. Specifically, the surfaces forming the gap were selected for the type A specimens, and the grooved surface was chosen for the type B specimen. In order to improve the image quality, the specimen surfaces were first coated with a gold film and then observed in a scanning electron microscope (JEOL JSM-5610LV, eBay Inc., San Jose, CA, USA). Elemental composition was derived from energy dispersive X-ray fluorescence spectra (EDX). Crystalline phases were analyzed by X-ray diffraction (D8, Cu Kα; Bruker, Billerica, MA, USA), operated at a voltage of 40 kV and at a scanning step of 6°/min.

## 3. Results and Discussion

**Figure 2:**
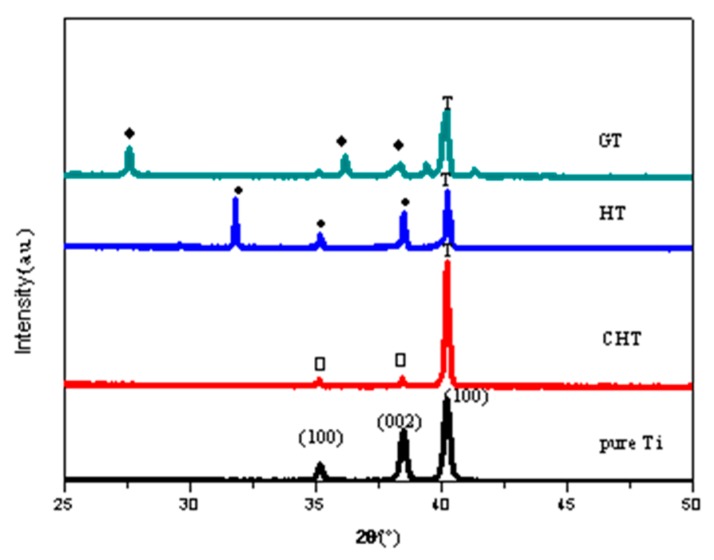
XRD patterns of different specimens: pure Ti, CHT, HT, and GT (T: Titanium; □: Anatase; ♦: Rutile; •: Ti_4_O_7_).

Figure 2 shows XRD patterns of different specimens. For the untreated high purity titanium, the three peaks at 35°, 38°, and 41° was attributed to the (100), (002), and (100) diffractions of pure titanium, respectively. For CHT, the same three peaks at 35°, 38°, and 41° were observed, but the intensities of the peaks were different than for the untreated high purity titanium: The peak at 41° grew in intensity, whereas the other two peaks were reduced. This could be caused by chemical treatment with hydrogen peroxide. Titanium oxides were not identified for CHT, indicating that the amount of titanium oxides formed during thermal oxidation at 400 °C for 1 h may be too small to be detected by XRD. From the XRD patterns for HT, the thermal oxidation at 400 °C for 5 h with the specimen being in contact with the Na_2_O·2B_2_O_3_ powder promoted the generation of Ti_4_O_7_, a kind of metastable titanium oxide. This implied that in addition to holding time, the Na_2_O·2B_2_O_3_ powder played an important role for the formation of titanium oxides. In GT, rutile was the dominating phase. This was apparently due to the high temperature treatment at 600 °C for 5 h. 

**Figure 3:**
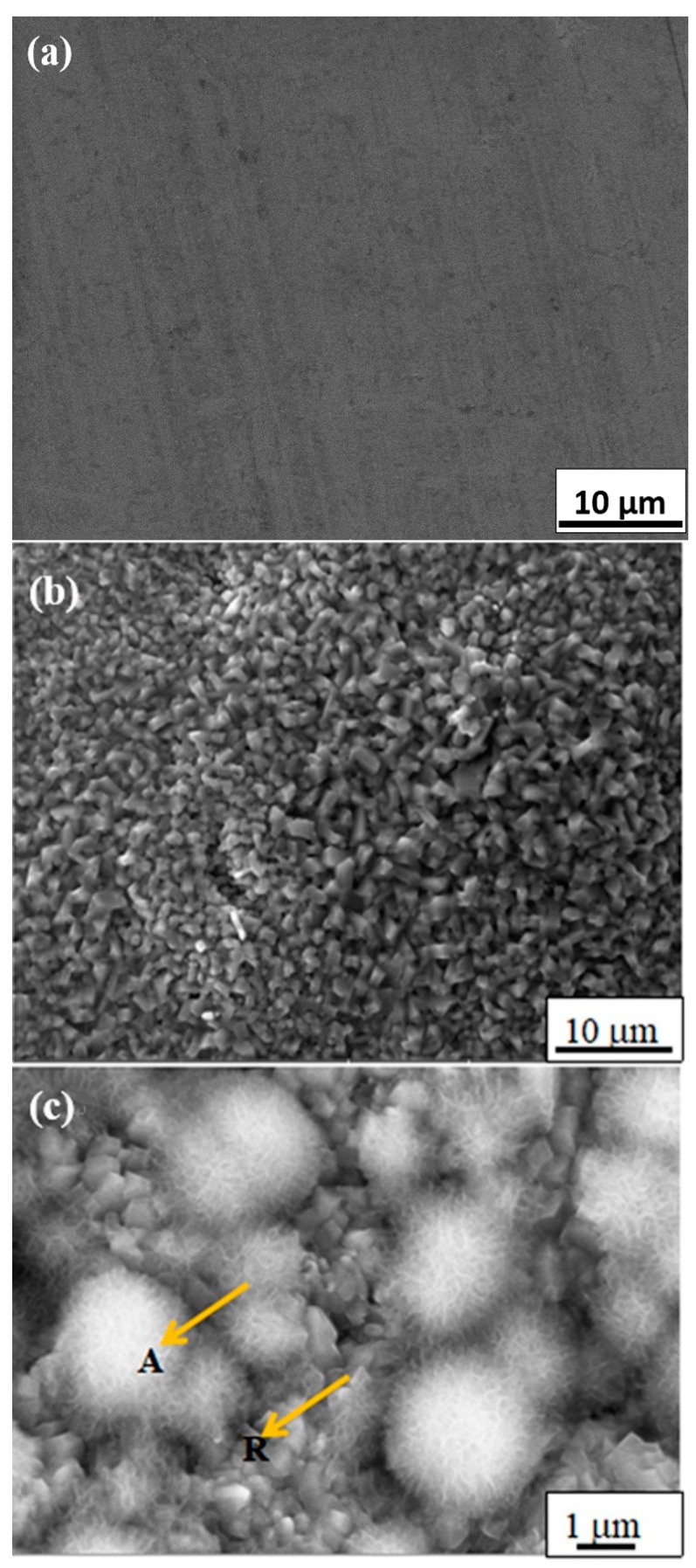
SEM on surface of GT: (**a**) No apatite on surface of pure titanium after soaking in SBF for five days; (**b**) Rutile crystals on surface of GT after heat treatment at 600 °C for 5 h; (**c**) Apatite on surface of GT after soaking in SBF for five days (pH 7.4, 36.5 °C).

Studies have shown that surface topography—such as coating roughness, thickness, pore size and distribution—would affect the bioactivity of implants and cell behaviors such as attachment, proliferation, and differentiation [18]. It is reported that the increase of the surface roughness of the titanium alloy is beneficial to the proliferation and diffusion of osteoblasts [19]. Some in vitro experimental studies [20,21,22] also demonstrated that the attachment of osteoblastic cells was enhanced on submicron scale structures but not on smooth surfaces. Nano-structured titanium oxide is more favorable for the proliferation and differentiation of cells [23]. In general, well-developed filopodia directly entered nanometer-sized pores for the initial attachment of the osteoblastic cells. Micro- and nano-structured TiO_2_ coatings, which can significantly promote cell proliferation and adhesion, have become the research focus in the field of biomedical titanium alloy for a period of time [24,25]. In our study, the surface morphologies of GT before and after soaking in SBF are shown in Figure 3. After heat treatment at 600 °C for 5 h, needle-like crystals with a diameter of about 1~2 µm were prevalently found (Figure 3b) and determined to be rutile crystals according to the X-ray diffraction (XRD) analysis (Figure 2). Our results indicated that rutile-type titania, formed at 600 °C for 5 h, was regularly arranged with uniformly distributed nanometer scale pores, which increased the surface roughness and specific surface area. Albrektsson et al. [26] reported that a moderate roughness of 1~2 µm may limit mechanical interlocking between the implant surface and bone for growth. An increase in peri-implants, as well as an increase in ionic leakage, results from high surface roughness [27]. Based on their findings, the porous rutile-type titania in this study should be perfect for human implants. The morphology of rutile-type titania crystals in this work is similar to that in human bone, which would make it much more biocompatible.

**Figure 4:**
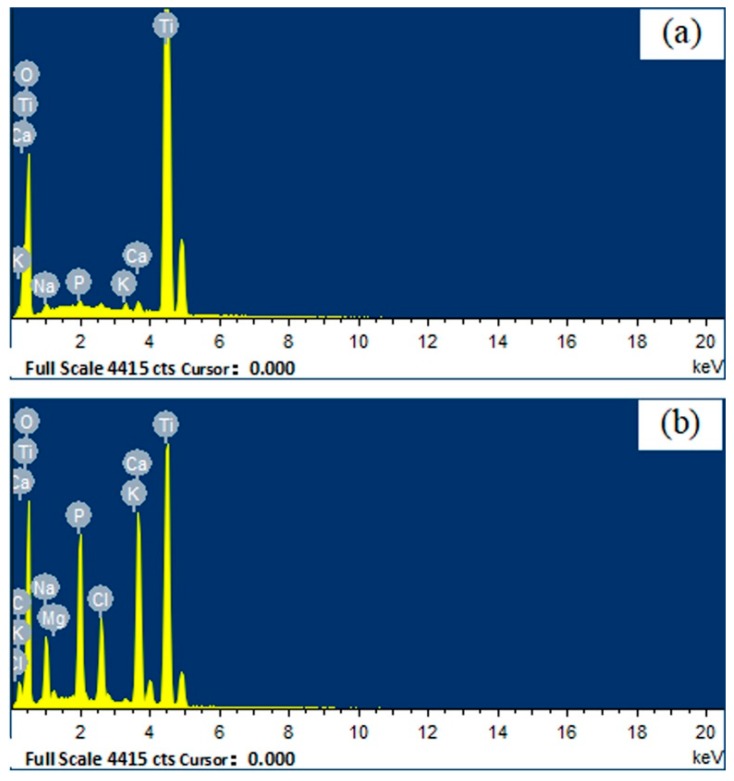
EDS of different point on surface layer of GT (**a**) EDS of point R on surface layer of GT; (**b**) EDS of point A on surface layer of GT (R: rutile-type titania substrate; A: apatite).

It was reasonable that Figure 3a did not show any apatite formation on the surface of pure titanium without surface modification after soaking SBF solution for five days, because no proper oxide layers were formed. Figure 3c shows the SEM photographs of GT surface after soaking in SBF at 37 °C for five days. It can be seen that an apatite layer with many microspheres had formed. At a high magnification and enhanced contrast, we can distinctly find many spherical apatite agglomerates with fine hairy crystals, which should have excellent bone conductivity and cell adhesion [28]. EDS analysis for selected areas A and R (see the arrows in Figure 3c) showed that only little amount of Ca and P was present in area R free of apatite (Figure 4a, Ca 0.50%, P 0.23%), whereas the spherical apatite with fine hairs (area A) showed the presence of much more Ca and P (Figure 4b, Ca 10.10%, P 6.33%). The Ca/P ratio of apatite is 1.58, very close to the 1.67 reported in literature [29]. As for the Cl and Mg ions detected by EDS, they must be from residue of SBF which contains a large amount of these ions.

**Figure 5:**
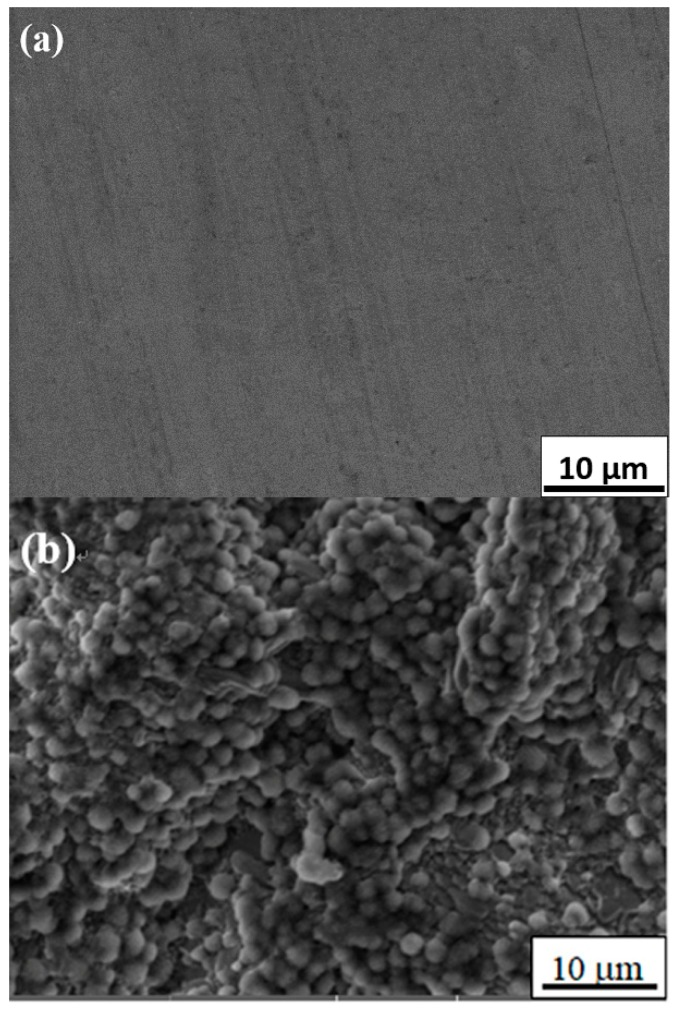

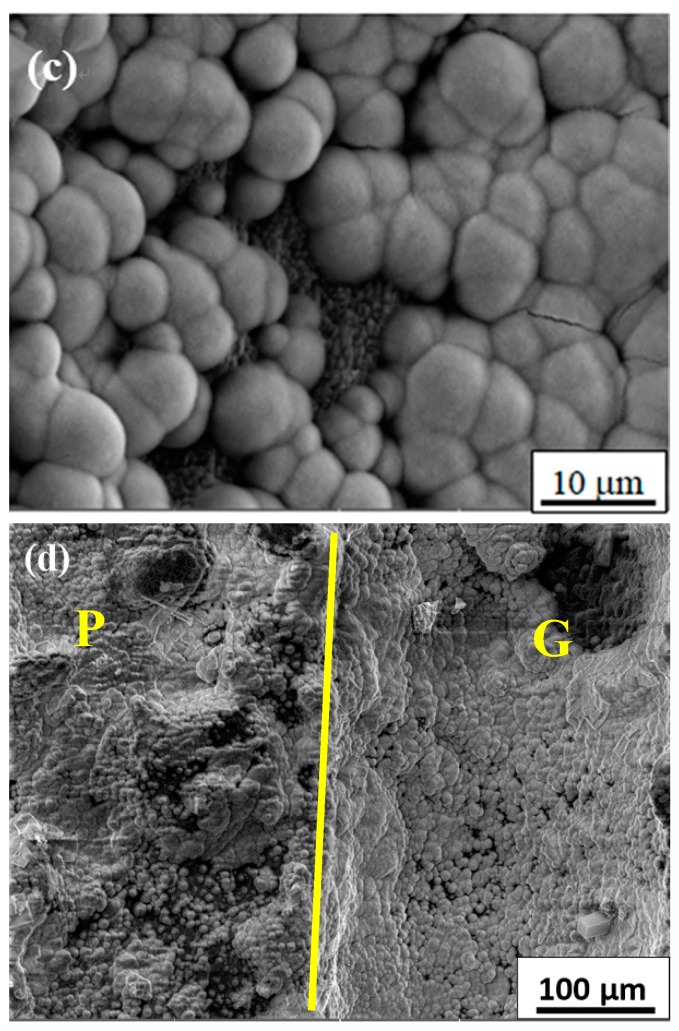
SEM micrographs of surface layer of pure titanium and GT after immersion in SBF at 37 °C for different days ((**a**) pure titanium immersed for 15 days; GT immersed for (**b**) 5 days; (**c**) 14 days; (**d**) dense apatite particles in the platform (P) and on the grooves (G); 14 days in SBF).

We did not find the deposition of apatite on the surface of pure titanium after soaking it in SBF solution for 14 days (see Figure 5a), either. In SBF, the white coating can be observed by the naked eye after five days and it grew thicker with aging for about two weeks. Unlike previous studies [6,7,8,9], we found apatite formation not only in the grooves, but also on the platform as dense apatite particles (see Figure 5d). This finding showed that high-purity titanium was more bioactive than the other samples. Figure 5 compares SEM surface micrographs of GT after immersion in SBF at 37 °C for 5 days (Figure 5b) and 14 days (Figure 5c). Figure 5b showed that the initial nucleation of apatite proceeded individually. The diameter of each microsphere is approximately 2 µm to 3 µm. The newly formed apatite showed a furry, cluster-like shape. After immersion in SBF at 37 °C for 14 days, apatite microspheres had grown up and become denser with time. When they grew to a diameter of about 10 µm, coalescence took place and cracks appeared. Secondary nucleation may start on top of the primary apatite surface. At this time, apatite appears in shape of rounded pearls. 

**Figure 6:**
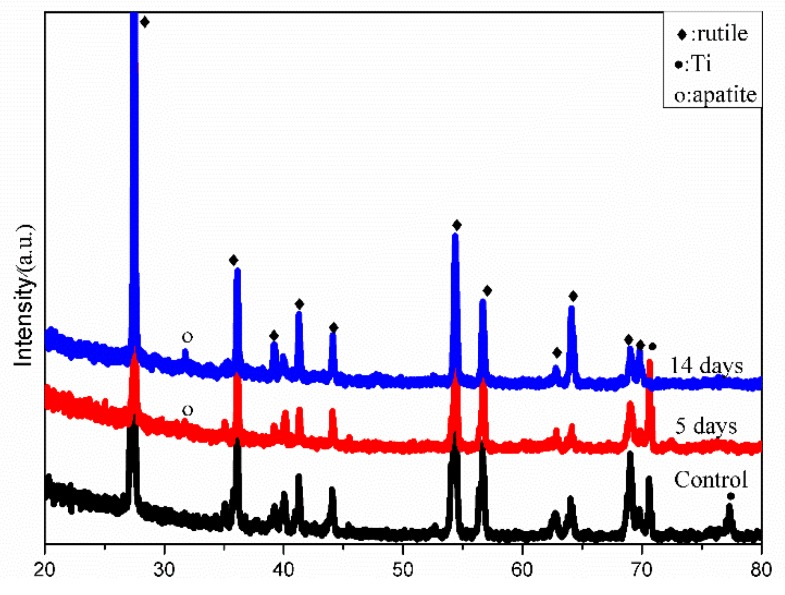
XRD patterns of GT before (control) and after immersion in SBF at 37 °C for 5 and 14 days.

The XRD profiles in Figure 6 indicated that the content of apatite gradually increases with the soaking time. The peak of apatite was observed at 32° after soaking in SBF for 5 days and 14 days. No other apatite peaks were observable, indicating the formed apatite is highly textured. Reagent of K_2_HPO_4_·3H_2_O provides HPO_4_^2−^. Crystallization of HA can be written in terms of Equation (2)

HPO_4_^2−^ = H^+^ + PO_4_^3−^ (1)

10Ca^2+^ + 6 PO_4_^3−^ + 2OH^−^ = Ca_10_(PO_4_)_6_(OH)_2_ (2)

In addition, the following reactions are also carried out in the solution

Ti − 4e^−^ = Ti^4+^ (3)

Ti^4+^ + O^2−^ = TiO_2_ (4)

Ti^4+^ + 2HPO_4_^2−^ = TiP_2_O_7_ + H_2_O (5)

TiP_2_O_7_ is deemed to have good bioactivity due to its hydrolysis into HPO_4_^2−^ ions according to Equation (5) [30] Apatite is only precipitated on bony tissue under normal conditions because the energy barrier for apatite nucleation is very high and is lowered only at the bony tissue site [12]. Therefore, this regularly arranged rutile-type titania with nanoscale pores provided more chemically active sites due to larger specific surface area. Those active sites induce the apatite nucleation. Once the apatite nuclei are formed, they spontaneously grow, consuming the calcium and phosphate ions from the surrounding SBF.

**Figure 7:**
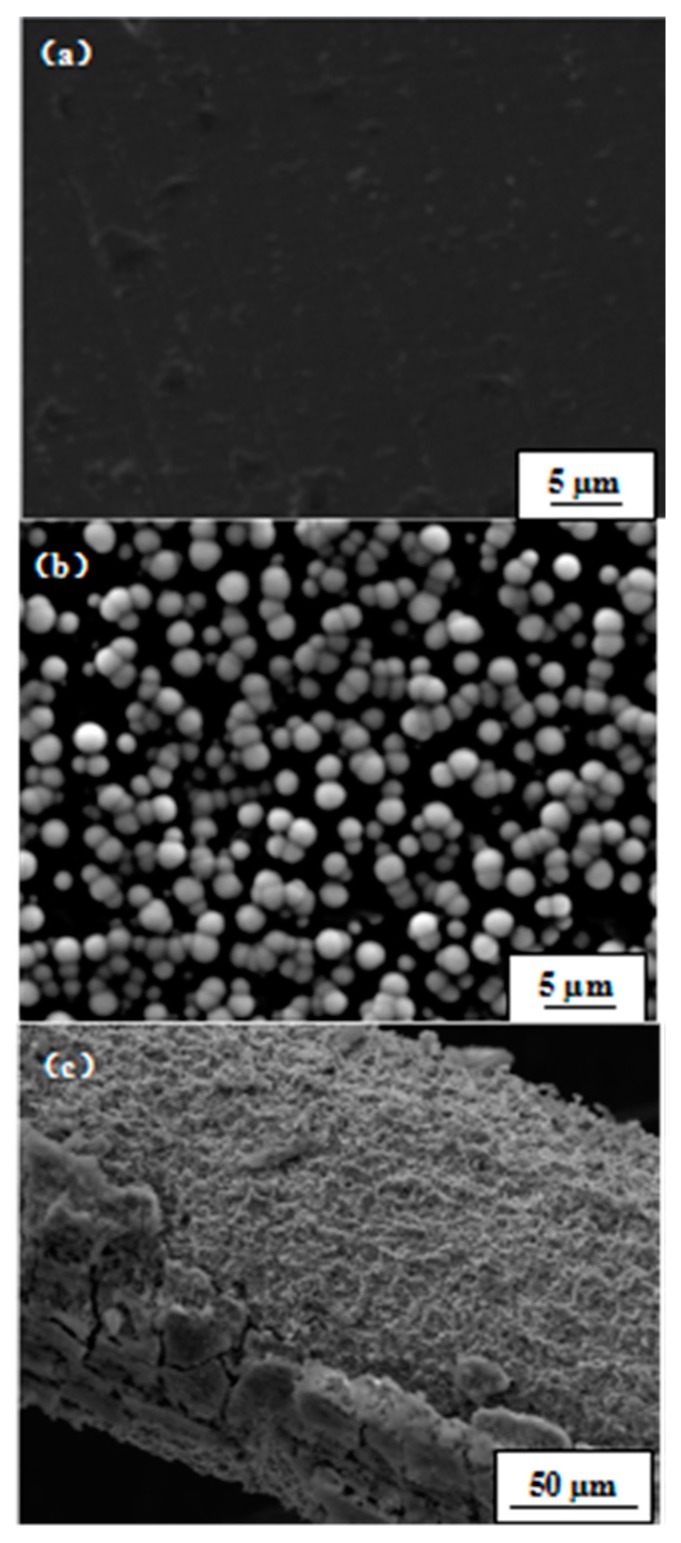

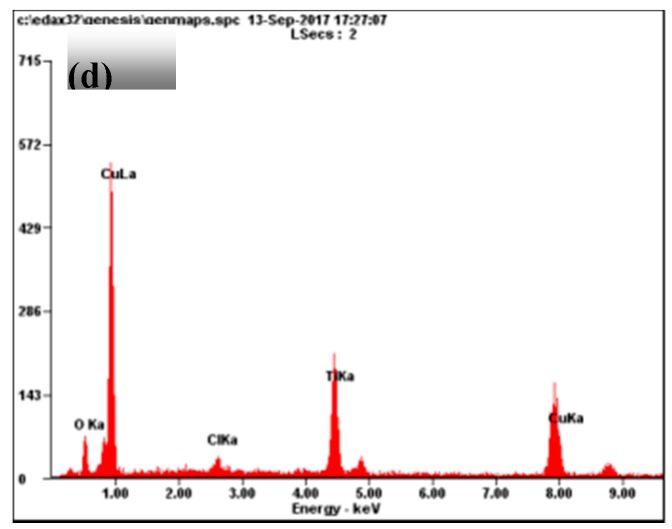
SEM photographs and EDS of HT after soaking in SBF for five days: (**a**) surface morphology of HT before soaking in SBF; (**b**)surface morphology of HT after soaking in SBF for five days; (**c**) surface morphology of copper wire; (**d**) EDS of point P on surface layer of HT.

**Figure 8:**
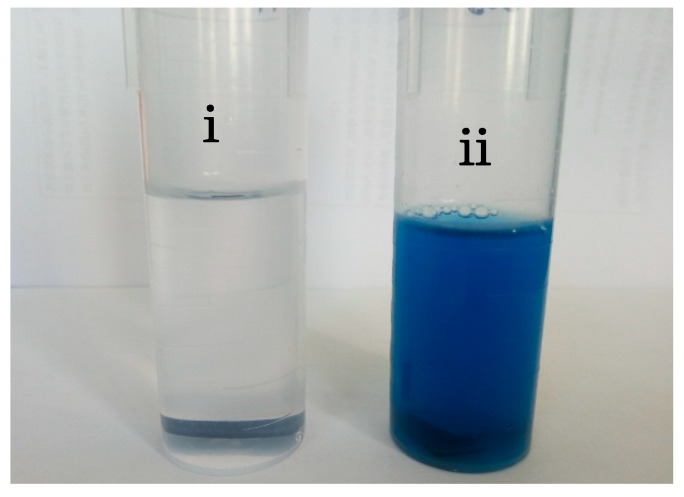
Color change: (i) without copper wire; (ii) with copper wire.

After soaking in SBF for five days, the surfaces of both CHT and HT were found to be covered by many spherical particles (Figure 7a), which have a diameter of about 1 µm and are evenly distributed. At first glance, these particles can be wrongfully taken as apatite particles as those shown in Figure 3c. Yet, a closer look reveals that the surface of these particles is much more smooth than apatite particles and without fine hairs, what is typical for apatite (see Figure 5b). To find out what these particles are, an EDS analysis was carried out. Figure 7c showed that these spherical particles are mainly composed of copper. Because we used copper wire to separate HT and CHT specimens (Figure 1) when soaking them in SBF, it is reasonable that these copper particles are a corrosion product of copper wire. Indeed in Figure 7b, the copper wire surface was corroded. About 5 µm long detached layer came off from the copper wire. The corroded area appears porous. In addition, the originally colorless SBF liquid gradually changed to a color of light blue with increasing soaking time, indicating the presence of Cu^2+^ complex ions (see Figure 8). All these signs indicate that the fine spherical particles on surfaces of CHT and HT are Cu particles. 

To our surprise, no apatite was found on surfaces of both CHT and HT. This must be due to corrosion of copper wire in our experiment. The presence of copper ions in SBF and copper particles on top of titania layer has obviously hindered apatite formation in our study. However, the mechanism by which copper inhibits biological activity is not well understood in this article. To avoid such a ‘copper poisoning’ effect, a corrosion resistant material should be used as spacer for separating the pretreated titanium specimens. In a similar experiment, Sugio et al. used titanium blosters to support heat treated titanium substrate [7], and they observed apatite-like microspheres on their titanium substrates after soaking them in SBF.

## 4. Conclusions

Based on the results obtained in this study, following conclusions can be drawn:

(1) Rutile-type titania formed on high-purity titanium possesses splendid bioactivity.
(2) A porous microstructure of rutile-type titania plays an important role in promoting apatite forming ability.
(3) The presence of copper ions in SBF and copper particles on top of the titania layer can hinder apatite formation.

## Figures and Tables

**Table 1:** Basic element content of high purity Titanium (99.99%).

Element	Ti	Al	P	V	Cr	Fe	Ni	Cu	Zn
content (ppm)	substrate	4.577	4.540	1.122	3.992	200.113	14.541	2.329	1.166

**Table 2:** Different chemical/heat treatments of specimens.

Sample		Size (mm^3^)	Treatment Method
Type A	CHT	10 × 20 × 1	Chemically treated with 30% wt H_2_O_2_ at 80 °C for 3 h, then heated at 400 °C for 1 h
	HT		Heated at 400 °C for 5 h covered with 50 µm Na_2_O∙2B_2_O_3_ powder
Type B	GT	10 × 10 × 3	Machined micro-grooves of 50 µm both in depth and in width, then heated at 600 °C for 5 h
